# AI-Assisted Computed Structure Models for Pre-Ubiquitylation Complexes Assembled by Respiratory Syncytial Viral Suppressors of Cellular Interferon Response

**DOI:** 10.3390/ijms27052437

**Published:** 2026-03-06

**Authors:** Sailen Barik

**Affiliations:** Independent Researcher, 8220 Walter Court, Mobile, AL 36695, USA; barikfamily@gmail.com

**Keywords:** Alphafold 3, AI models, ubiquitin, proteasome, nonstructural proteins, RSV, interferon, STAT2

## Abstract

Multiple viruses suppress the antiviral defense system of the host for optimal growth and pathogenesis by co-opting the ubiquitin-mediated proteasomal system (UPS) that promotes the degradation of cellular substrates belonging to the interferon pathway. In the *Orthopneumovirus* genus, respiratory syncytial virus (RSV), a significant pathogen in human and other animals, employs a pair of viral nonstructural proteins (NS1, NS2) to assemble the UPS. The lack of experimental three-dimensional structures of the substrate proteins and the NS-assembled UPS has impeded progress in our understanding of the mechanism of this assembly process. In an effort to remedy this deficiency, I have taken advantage of the burgeoning field of AI (artificial intelligence) and machine learning programs, such as AlphaFold3, to model the pre-ubiquitylation cores in various combination of the subunits to construct three-dimensional structures, named ‘computed structure models’ (CSMs). The UPS core universally comprises an adapter protein connected to the “substrate” that is to be degraded by the “substrate receptor”. The NS proteins are believed to act as receptors, and cellular Elongin BC as an adapter. These CSMs lend support to the biochemical results where known while also suggesting that the complete core of three proteins is energetically more stable than a complex of only the NS protein and the substrate. In the absence of experimental structures, these results offer, for the first time, a mechanistic insight into RSV-triggered assembly of the UPS, which should allow for a better design of future experiments, and eventually new antiviral regimens.

## 1. Introduction

Upon being infected by pathogens, viruses in particular, the host cells activate the type I interferon (IFN) pathway, which then acts as antiviral [1,2,3,4]. In counter-response, the viruses have evolved specialized functions that suppress this pathway [5]. Several laboratories including ours have been studying the mechanism of IFN suppression by RNA viruses, particularly in respiratory syncytial virus (RSV) that causes major respiratory illness in human neonates, sometimes causing death and often leading to asthma in the adulthood [6,7].

Following viral infection, the cellular IFN gene is induced by a cascade signaling pathway [1,8,9], resulting in the activation of IRF3 and IRF7, transcription factors that are essential for the induction of IFN genes. The liberated IFN binds to its cognate receptors and triggers the IFN response (signaling) pathway, whereby two other transcription factors, known as Signal Transducer and Activator of Transcription (STAT1 and STAT2), are activated. The two STATs, together with IRF9, forms a heterotrimer, known as IFN-Stimulated Gene (ISG) Factor 3 (ISGF3), which enters the nucleus and induces a large family of ISGs. Many ISGs function as antivirals that confer resistance to the virus, allowing for virus replication [1,2,3,4]. Thus, a better understanding of IFN suppression and its inhibition is an important goal in the therapy of viral diseases.

Early studies from several laboratories, including ours, demonstrated that the two nonstructural proteins of RSV (NS1 and NS2) act as essential antagonists of the host type I interferon (IFN), facilitating virus growth by suppressing IFN induction and response. Notably, recombinant RSV lacking NS genes (ΔNS1/ΔNS2) show significantly higher IFN production, and lower replication rates in IFN-proficient cells and in animals, but improved growth in IFN-deficient cells [10,11,12,13,14]. Regarding the mechanism, others and we showed that the NS proteins degrade multiple proteins of the IFN pathways [10,11,12,13,14,15,16,17]. Subsequently, we reported that NS proteins of another orthopneumovirus, namely the Pneumonia Virus of Mice (PVM), also degrade many members of the IFN pathways in the murine cell [18,19]. Of the two STAT proteins that form ISGF3, we have been interested in STAT2 since it appears to be more important than STAT1. For example, cells that are genetically deficient in STAT1 but overexpress STAT2 supported IFN-induced expression of key antiviral ISGs and inhibited the growth of RNA viruses such as encephalomyocarditis virus (EMCV) and vesicular stomatitis virus (VSV) [20]. The STAT2–IRF9 complex, devoid of STAT1, also plays an important role in the signaling by type II IFN—namely, IFNγ [21,22,23,24]. Other examples include enhanced VSV virulence in STAT2 knockout mice [25] and increased pathogenesis of Rift Valley fever [26] and Crimean–Congo hemorrhagic fever viruses [27]. Many viruses, therefore, have evolved to target STAT2 for IFN suppression, and this is also true of RSV and PVM. Multiple lines of biochemical evidence revealed that the degradation is catalyzed by the ubiquitin-mediated proteasomal system (UPS), assembled by the NS proteins of these viruses [10,16,17,19]. However, the molecular and structural details of the assembly process have remained unexplored, even in human RSV, which has received greater attention. The lack of progress in this field is largely due to the absence of higher order structures of the participating proteins. The problem is particularly acute with PVM, since the structures of its NS proteins or the mouse equivalent of the UPS proteins have remained unsolved.

Over the past few years, several approaches that use artificial intelligence (AI) and machine learning have been developed to successfully generate ‘computed structure models’ (CSMs) of proteins from amino acid sequences [28,29,30,31,32,33]. In the absence of crystal structures of NS-UPS complexes, I have employed several of these techniques to produce the CSMs for viral NS-assembled UPS cores to have a glimpse of how they may interact. Here, the terms “model” and “CSM” have been used synonymously and interchangeably.

## 2. Results

### 2.1. Modeling of NS-STAT2 Complexes

The AI-based program AlphaFold3 (AF3 for short), which represents a major advance in protein structure prediction, was used in the majority of work performed here for generating three-dimensional (3D) structures of single proteins and multiprotein complexes since this study required both [28,29].

The full structure of the virally assembled UPS complex is not known for either of the two orthopneumoviruses currently known, i.e., RSV and PVM. Since the NS proteins constitute a large proportion of our studies, our first step was to know their structures and test the reliability of the predicted ones. A single crystal structure was recently published for each of these proteins: NS1, PDB 5VJ2 [34], and NS2, 7LDK [35]. However, the oligomeric status of the proteins has been debated. The NS2 crystal consists of asymmetric units, each comprising three monomers that are slightly different in conformation, such that they were designated A, B, and C. However, solution studies indicated the NS2 is largely a monomer. NS1, in contrast, was found to be a trimer of identical conformation in the crystal. It appears that both NS proteins have a metamorphic nature that may respond to environmental triggers, including crystallographic conditions and the presence of interacting proteins. Previously, we constructed recombinant NS1 and NS2 that were tagged with FLAG and HA epitopes and expressed them by transient transfection in all combinations [12]. Co-precipitation results and relative mobility in denaturing gel revealed that NS1 and NS2 form homo- and heteromers (i.e., NS1-NS1, NS2-NS2, and NS1-NS2), although the stoichiometry of the proteins in the complexes or their activities could not be determined. No trimer was detected.

**Figure 1:**
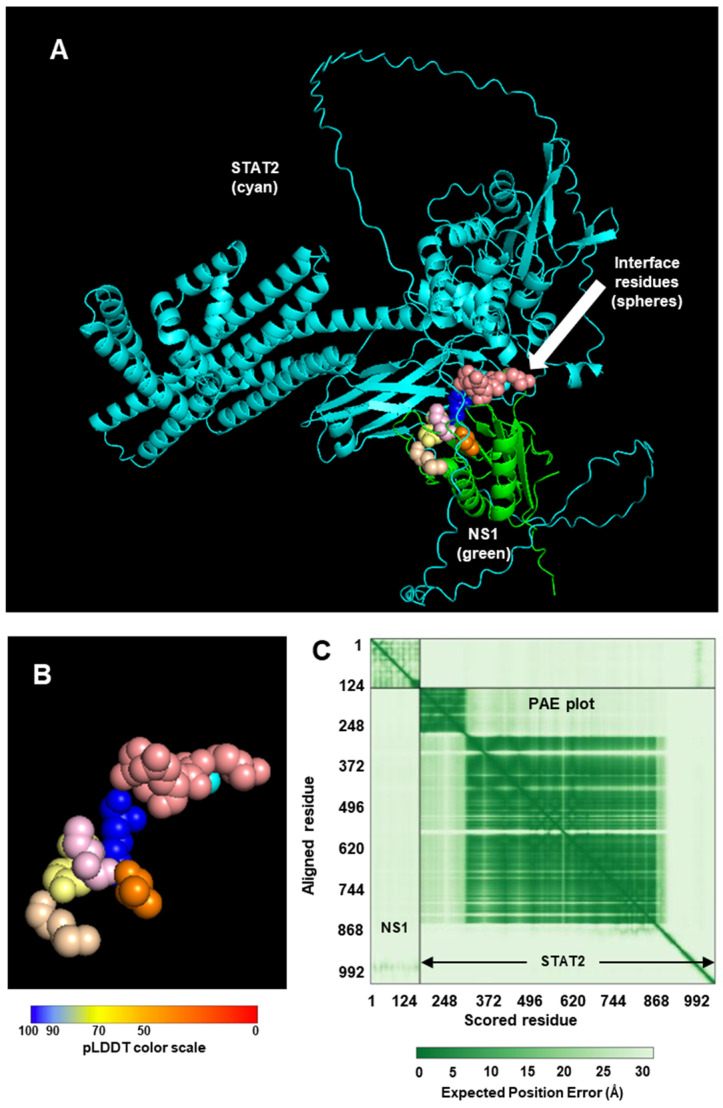
Heterodimeric complex between RSV NS1 and human STAT2. (**A**) The 1:1 STAT2:NS1 complex, created by AlphaFold3 (https://alphafoldserver.com/) (accessed on 1 December 2025) [28], is depicted in PyMol (www.pymol.org) (accessed on 15 November 2025) [36]. STAT2 = cartoon in Cyan; NS1 = Green. The interface amino acid residues were determined by their distance in PyMol display [38], which was also supported by their interaction energy values, determined in the INTAA site [39,40]. These residues, shown as spheres and boxed with dotted line, are colored in the commonly used pLDDT color range that is presented under panel (**B**): dark blue (very high confidence, >90), light blue (confident, 70–90), yellow (low confidence (50–70), orange (very low confidence, <50). (**B**) Enlarged view of the interface area, boxed in Panel (**B**). (**C**) Predicted Aligned Error (PAE) in green, viewed in PAE viewer (https://pae-viewer.uni-goettingen.de) (accessed on 2 March 2026) [41]. The NS1 and STAT2 areas are so marked. The graded color gradient of the error scale is shown underneath. By usual convention, a dark green tile designates a strong prediction (low error), and light green tile indicates weak prediction (high error). Note that the NS1 quadrant comprises mostly low-confidence predictions, whereas the STAT2 quadrant enjoys high confidence.

**Figure 2:**
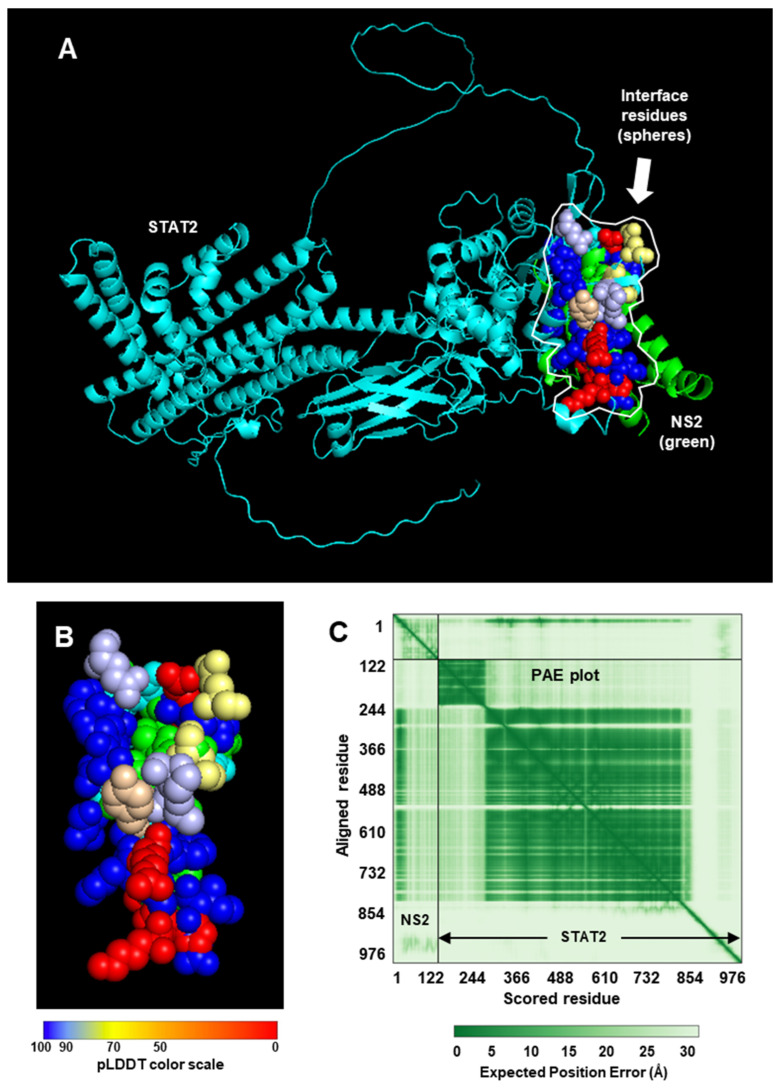
Heterodimeric complex between RSV NS2 and human STAT2. The strategy is the same as in Figure 1, only the NS1 was replaced by NS2. (**A**) The STAT2:NS2 complex was generated by AlphaFold3, and is depicted in PyMol. Color codes are also identical, i.e., STAT2 = cyan; NS2 = green. The interface residues are boxed in panel (**A**), and colored in the pLDDT color range. (**B**) Enlarged view of the interface area. (**C**) PAE plot with the NS2 and STAT2 areas marked. Note that the plot that is nearly the NS2 quadrant comprises mostly low-confidence predictions, whereas the STAT2 quadrant enjoys high confidence.

Since our goal here is to construct CSMs of NS proteins complexed with UPS factors (e.g., STAT2, Elongin C, etc.), I decided to start with the monomer of the NS proteins, regardless of the oligomeric nature of the protein in the crystal. In the RCSB structure bank, the STAT2 structure is only available in complex with small molecules or other proteins (such as Zika viral IFN suppressor, NS5, whose sequence is very different from the RSV or PVM NS proteins) [36,37]. The NS structures were, therefore, complexed with an AF3-created STAT2 structure (Figure 1 and Figure 2).

We will draw the following conclusions from these observations since similar analyses and presentations have been used for other interactions in this paper. As seen for both NS1 and NS2, the PAE is high, which means AF3 has low confidence in its prediction of the alignment of the subunits. Other metrics of accuracy are the Template Modeling (TM) scores: pTM, which measures the quality of the whole structure by assessing the similarity between the predicted structure and the hypothetical true structure; and iPTM, which focuses on the interface quality of the modeling. The iPTM is actually derived from the TM score, using the PAE matrix, but only considers the amino acid pairs that interact between the two chains (subunits). The pTM and iPTM, provided by AF3 along with the structure of the two complexes, are: pTM 0.62, iPTM 0.70 for NS1-STAT2, and pTM 0.64, iPTM 0.67 for NS2-STAT2. The iPTM values in this range (0.6–0.7) suggest that the prediction falls into a “gray zone” where the interface could be correct, partially correct, or wrong. The pTM values suggest a reasonable, if not high-confidence, prediction of the overall structural arrangement of the complex.

**Figure 3:**
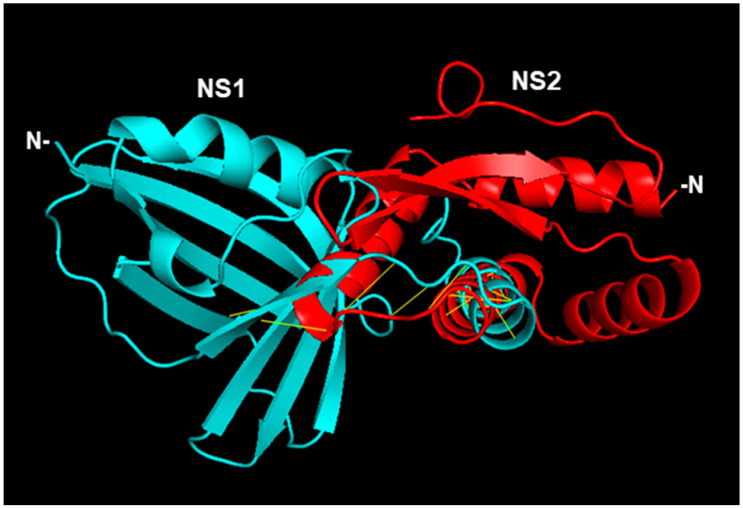
Dissimilarity between the 3D structures of the RSV NS1 and NS2 proteins. The complex was created in PyMol, using the monomers from the crystal structures. The N-terminal ends are indicated. The yellow lines, which connect the interactive amino acids in the two helices and a loop, are ~3.5 Angstrom apart, dictated by PyMol’s default distance cut-off.

To assess the stability of the complexes, I obtained the Kd (equilibrium dissociation constant) values using PRODIGY [42,43,44], from which Gibbs free energy change (∆G) was also calculated. A lower Kd indicates a more stable complex or higher affinity between the proteins; conversely, a higher Kd means a weaker complex or lower affinity. The Kd values were: NS1-STAT2, 1.2 × 10^−7^ M (=∆G −9.4 kcal/mol); and NS2-STAT2, 4.3 × 10^−11^ (=∆G −14.1 kcal/mol). The Kd values indicate that the NS1-STAT2 complex will form but the NS2-STAT2 complex may be significantly more stable; in other words, it will take a much lower concentration of NS2 and STAT2 for 50% of the NS2 and STAT2 to bind each other. Regarding the structural interactions, as mentioned earlier, NS1 and NS2 are quite dissimilar in their primary structures. However, since they both interact with STAT2 and degrade it with UPS, I tested the possibility that their higher order structures may be similar. It has long been known that nonhomologous proteins of very different amino acid sequences (with sequence identity as low as 3%) can fold into similar 3D structures due to convergent evolution in structure, since the folding is dictated by interactions between key residues in strategic places and other biophysical constraints [45,46]. In fact, in a large number of cases, the functions of novel proteins can be successfully predicted on the basis of known protein structures [47]. Nonetheless, alignment of the monomers in their crystal structure revealed no similarity (Figure 3).

**Figure 4:**
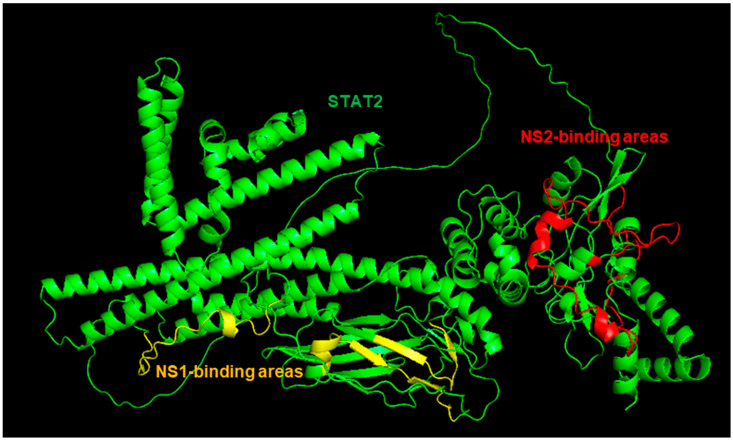
The overall binding areas of NS1 and NS2 on STAT2. The colors are as follows: STAT2, green; NS1-binding area, residues ~798–820 of STAT2, yellow; NS2-binding area, residues ~674–809 of STAT2, red. Note that the two binding areas appear closer in Figure 2 and Figure 3 because the structure in this figure was rotated to offer a better view of the distance.

Minor NS1-NS2 interactions between two closely spaced helices and a loop (Figure 3) involved the side chains of approximately eight amino acid residues. I would consider this irrelevant for STAT2 binding since NS1 and NS2 3D structures bind very different areas of the STAT2 structure that are distant from each other, as marked in Figure 4.

As implied in Figure 1 and Figure 2, the NS proteins do not touch all the residues in the areas marked in Figure 4, but rather specific ones that are not individually labeled for simplicity.

### 2.2. “Positive Control” and “Negative Control” of NS2 Interaction

**Figure 5:**
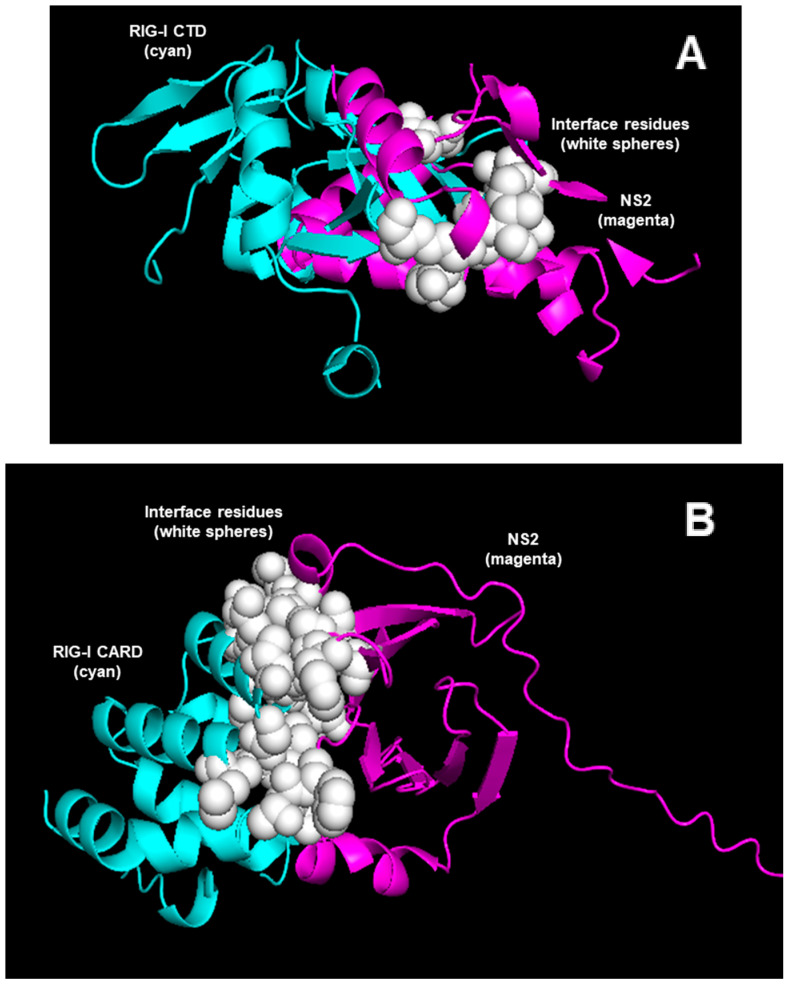
Alphafold 3-predicted structures of NS2, complexed with the 89-aa long CTD (**A**) or 131-aa long CARD (**B**) of RIG-I. The sequences can be found in the Supplementary Materials. The colors and labels are self-explanatory. As in the other CSMs generated in this paper, these models also show AF3-predicted structural interaction. The interface amino acid residues (unlabeled) are shown as white spheres, but they were contributed by both proteins. The PAE plots are shown in the next figure (Figure 6).

There is no experimental demonstration that the NS1 or NS2 protein directly binds STAT2. To support the reliability of the structural analysis performed above, we needed polypeptide “controls” that have been shown to bind or not bind the NS proteins. The RIG-I (Retinoic acid-Inducible Gene–One) protein is a cellular RNA-dependent helicase that acts as a sensor of infecting RNA [48], such as the early transcripts of RSV [49]. The binding then triggers the activation of the IFN induction pathway. RSV NS1 and NS2 also destroys RIG-I and this requires cellular UPS. Binding of RNA to RIG-I activates RIG-I through an intricate mechanism that involves a coordinated structural change in the protein [50,51], which I will describe here very briefly. In the absence of any RNA ligand (e.g., in the uninfected cell), RIG-I adopts an autoinhibited conformation, where the N-terminal CARDs (Caspase Activation and Recruitment Domains) are sequestered from interactions with other CARD-containing cellular molecules. Binding of viral RNAs to the CTD (C-Terminal Domain) changes the folding of RIG-I, releasing the N-terminal CARDs and relieving autoinhibition. Pei et al. [35] showed that NS2 directly binds the CARD domain but not the CTD. For example, their co-immunoprecipitation experiments using epitope-tagged CTD and NS2 as well as peptide-binding studies did not reveal NS2 interaction with this domain. I reasoned that the same might be seen in our structural analysis. To test it, I had the two domains folded by AF3 and conducted the complex formation and analysis as before, and present the structures here (Figure 5).

**Figure 6:**
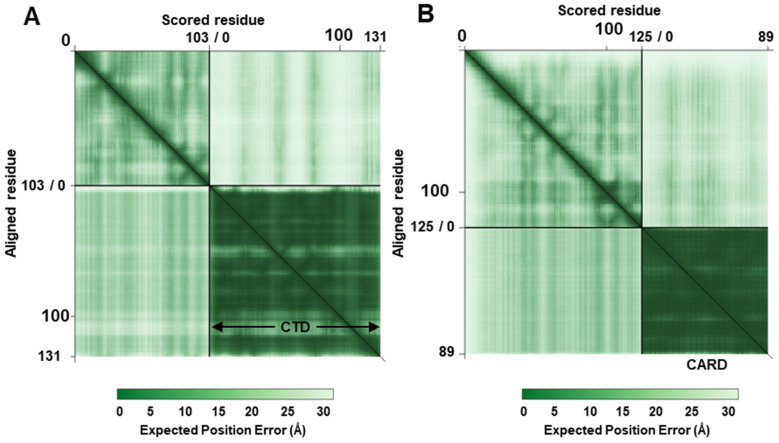
PAE plots with the CTD (panel (**A**)) and CARD areas marked. As in previous plots, the NS2 quadrant received low-confidence pLDDT scores. In panel (**B**), the 22-residue long flexible sequence of NS2 was removed, which may be reflected in the slightly darker corner of the NS2 quadrant.

As we have seen with NS-STAT2 predictions, the predicted structure plots do not demonstrate the strength or stability of a complex, and so I determined the dissociation constant of the structure, where a clear difference was noted. The Kd of the NS2 complex with CTD, the nonbinder polypeptide, was 3.1 × 10^−7^ M, while that of the complex with CARD, the binder polypeptide, was 8.1 × 10^−10^ M, suggesting that the CARD complex is approximately 300-fold more stable. This can easily explain the observed difference between the two domains of RIG-I, reported before [35], and at the same time offers confidence in this thermodynamic parameter of a CSM.

### 2.3. RSV NS Proteins Make Contacts with Elongin C in CSM

In the current understanding of the E3 ligase complex, the NS proteins bind to the substrate STAT2 on one hand and the Elongin C subunit of the ElonginBC complex on the other. Thus, I next constructed the CSM for NS1-Elongin C and NS2-Elongin C to first see if they are stable. The procedure was essentially the same as that used this far, i.e., the two sequences were folded and complexed by AF3, and then evaluated.

**Figure 7:**
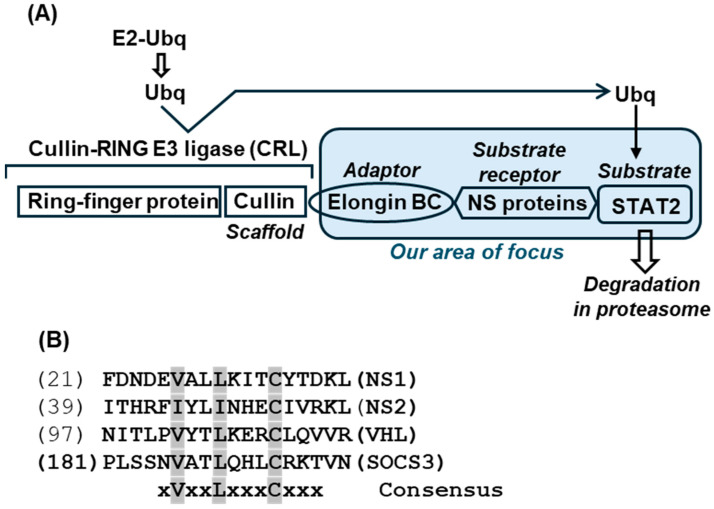
(**A**) The assembly of the core complexes of UPS. Ubiquitin (Ubq)-mediated proteasomal system (UPS) begins with the transfer of the ubiquitin moiety, activated by the activating enzyme E1, to the conjugating enzyme (E2). A ubiquitin ligase (E3 ligase) recognizes the specific target substrate, and facilitates the transfer of Ubq from the E2 to the substrate [53,54,55]. The most common type of E3 ligase belongs to the RING finger family, designated Cullin-RING E3 ligase (CRL), in which Cullin acts as a scaffold that brings the multiprotein complex together [56]. Cullin recruits Elongin BC (the adaptor), which connects to the substrate, bridged through a receptor. The Ubq serves as a tag that marks the substrate for degradation by the proteasome [57]. When the substrate is HIF-1α, the receptor is VHL; in the case of RSV, STAT2 is the substrate to be degraded, and the NS protein(s) likely serves as ‘receptor’ [17,58,59]. (**B**) BC-box consensus sequence in selected proteins. The putative BC-box in NS1 [17] and NS2 [60] are shown along with the classical BC-box in VHL and in SOCS3 (Suppressor of Cytokine Signaling 3) as two best-known examples [61,62]. In the consensus, the invariant Val, Lys, and Cys are highlighted. Note that this schematic does not reflect the three-dimensional shape of the proteins or the domains through which they make contacts with multiple other subunits.

I also used the UPS core assembled by VHL (Von Hippel–Lindau disease tumor suppressor) for comparison since it remains one of the best-studied complexes of this kind and often serves as the role model for other suppressors. In this complex, the VHL protein, acting as the ‘substrate receptor’, interacts with Elongin BC, forming the core complex (VHL-Elongin B/C) that acts as an E3 ubiquitin ligase to degrade the ‘substrate’ HIF-1α (Hypoxia-inducible factor 1-alpha), a process that is crucial for tumor suppression. Elongin B and C form a relatively stable heterodimer, with Elongin B binding to residue ~18–34 of Elongin C, which is essential to the formation of the functional Elongin BC complex [52] that then interacts with other proteins, such as VHL (or viral NS) via their BC-box motif (Figure 7).

**Figure 8:**
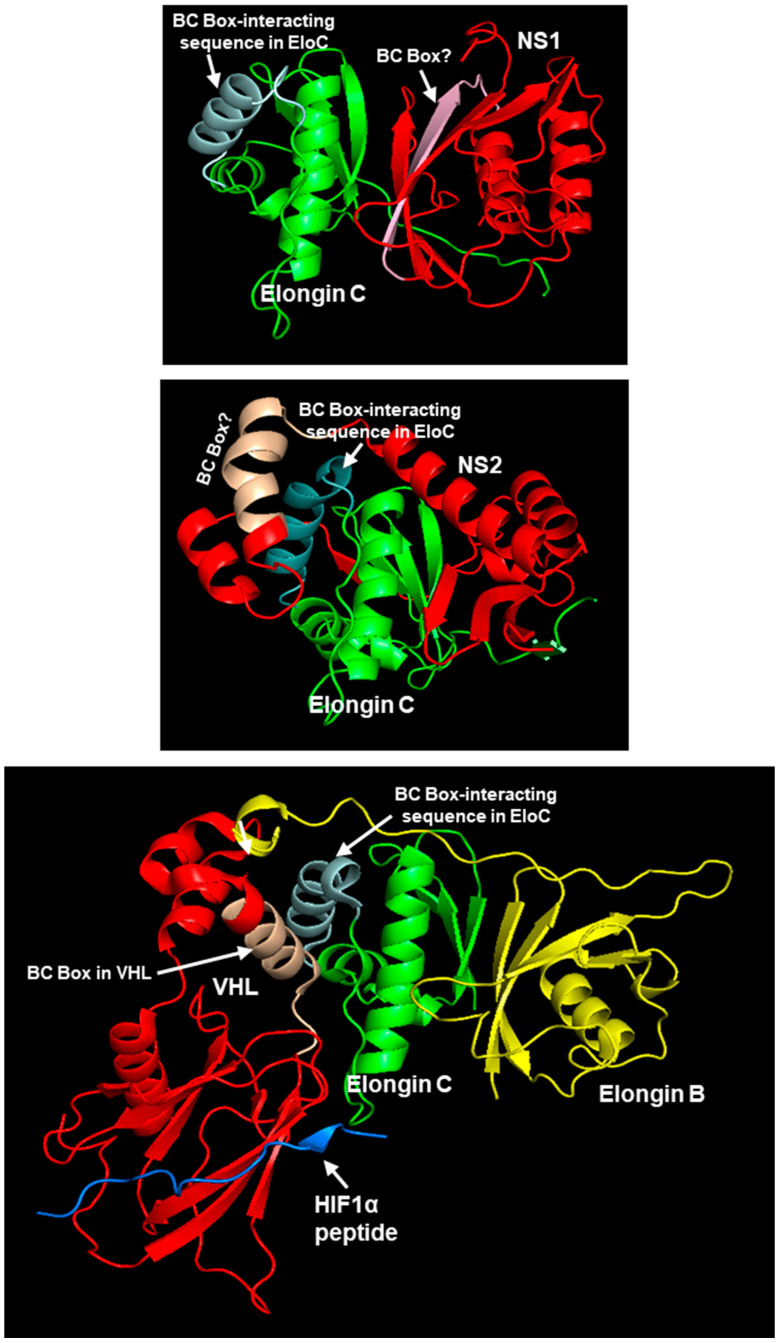
CSMs of Elongin C, complexed with either NS1 or NS2, are shown in the top two panels. The bottom panel displays the VHL complex derived from the crystal structure in PDB 1LM8. Most of the relevant items are labeled. Briefly, in all panels, Elongin C is green, and the substrate receptors (NS1/2, VHL) are in red; additionally, in the bottom panel, Elongin B is in yellow, and the short HIF-1α peptide is in blue. The BC-Box in the receptors, VHL/NS1/NS2, are also marked; while the box is well-established in VHL, those in NS1/NS2 remain speculative, indicated by the question mark [17,60]. Likewise, the interaction of the VHL BC-box with a specific Elongin C (EloC, for short) sequence has been documented [54]; this sequence is also marked in the NS1/NS2 panels, but its direct interaction with the putative NS1/NS2 BC-boxes remains questionable, as discussed later.

With the VHL complex in mind, the Elongin C-NS1 and Elongin C-NS2 models were created, and are shown here along with the quaternary complex of VHL (Figure 8).

Since the confidence of AF3 in the “control” NS2-RIGI-CARD and NS2-RIG-I-CTD complexes did not correlate with the strength or stability of the complex, whereas the dissociation constants (Kd) did, I also obtained the Kd values of these NS-EloC complexes. The values were 9.7 × 10^−8^ M for the NS1-EloC complex, and 9.3 × 10^−9^ M for the NS2 complex. Although the difference is only 10-fold, it does indicate stronger binding affinity of NS2 for EloC and higher stability of the NS2-EloC complex.

### 2.4. Model for the Trimeric Complex, Elongin C-NS2-STAT2

In the previous structures, the putative BC-boxes of both NS1 and NS2 appear to make contact with Elongin C, although parts of NS1 were located farther on the other side, and likely did not make contact with Elongin C. A large portion of Elongin C, consisting of two helices and three strands, appeared to be cradled in the prominent groove of NS2. The VHL protein, through its BC-box, made contact with yet another face of Elongin C, approximately resembling the position of one arm of the NS2 groove. The substrate peptide of HIF-1α is in direct contact with VHL, which is analogous to NS2 making contact with STAT2 (Figure 3). Of note, the Elongin B subunit of Elongin BC is on the other side of VHL. Thus, the individual proteins in the VHL complex are linked in the following order: Elongin B-Elongin C-VHL-HIF-1α. These structural results confirm that Elongin B may not play a direct role in the assembly process at this stage for either VHL [62,63,64] or NS2.

**Figure 9:**
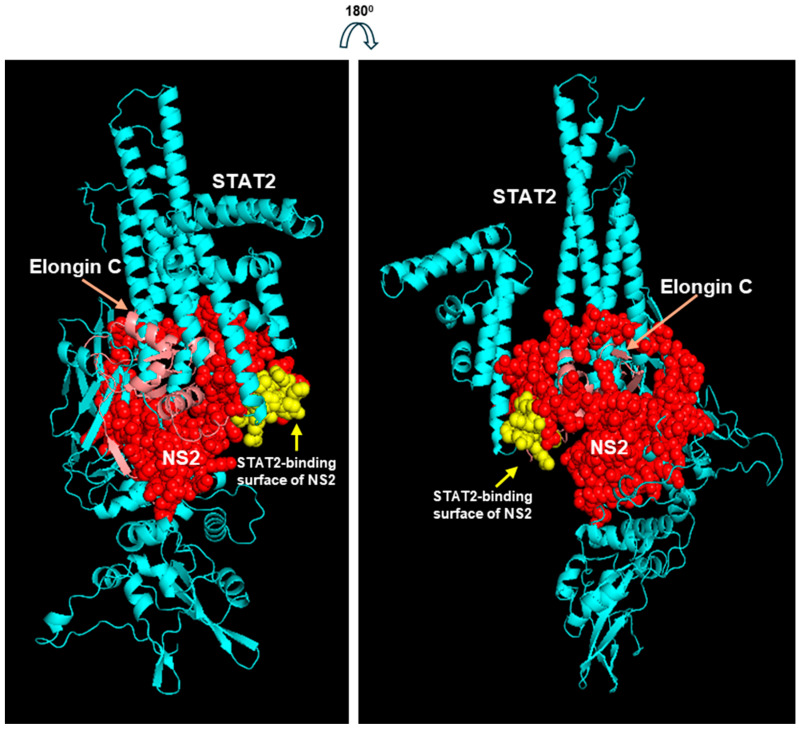
Bioinformatic structure of the RSV NS2-mediated E3 ligase core. The structures are colored and labeled: STAT2, cyan; NS2, red spheres, except its STAT2-interacting surface, which is in yellow; Elongin C, almost completely buried, is in light pink. Two views of the model are presented that are at 180° angle with each other (i.e., front and back view). Following the modeling, some of the large flexible loops of STAT2, which were not in contact with any structure, were deleted for a clearer view of the trimer.

Although AF3 provides high-accuracy predictions for many protein complexes, its performance can be less reliable for larger complexes [65,66]; moreover, for any program, the low confidence in each protein structure multiplies combinatorially. For this reason, I set a realistic goal of not adding EloB, but to just attempt to create the heterotrimeric Elongin C-NS2-STAT2 complex, since NS2 interacted with both of them. NS2 was chosen over NS1 for its somewhat stronger association in the previous heterodimers, and because its putative BC-box folded into a α-helix, resembling the classic BC-box of VHL, whereas that of NS1 was a β-strand (Figure 8). It was also hoped that the structure may reveal if BC-box interacts with any protein of the ligase complex. The trimer was made by Alphafold-Multimer in Chimera X, since it is considered more reliable for complexes than regular AF3 [57,58]; the highest confidence model is presented here (Figure 9). The predicted Kd of the heterotrimer was 2.1 × 10^−10^, ΔG = −13.2; thus, the trimer may be somewhat more stable than the NS2-Elongin C dimer with the Kd values in the 10^−8^/10^−9^ range (Supplementary Materials).

**Figure 10:**
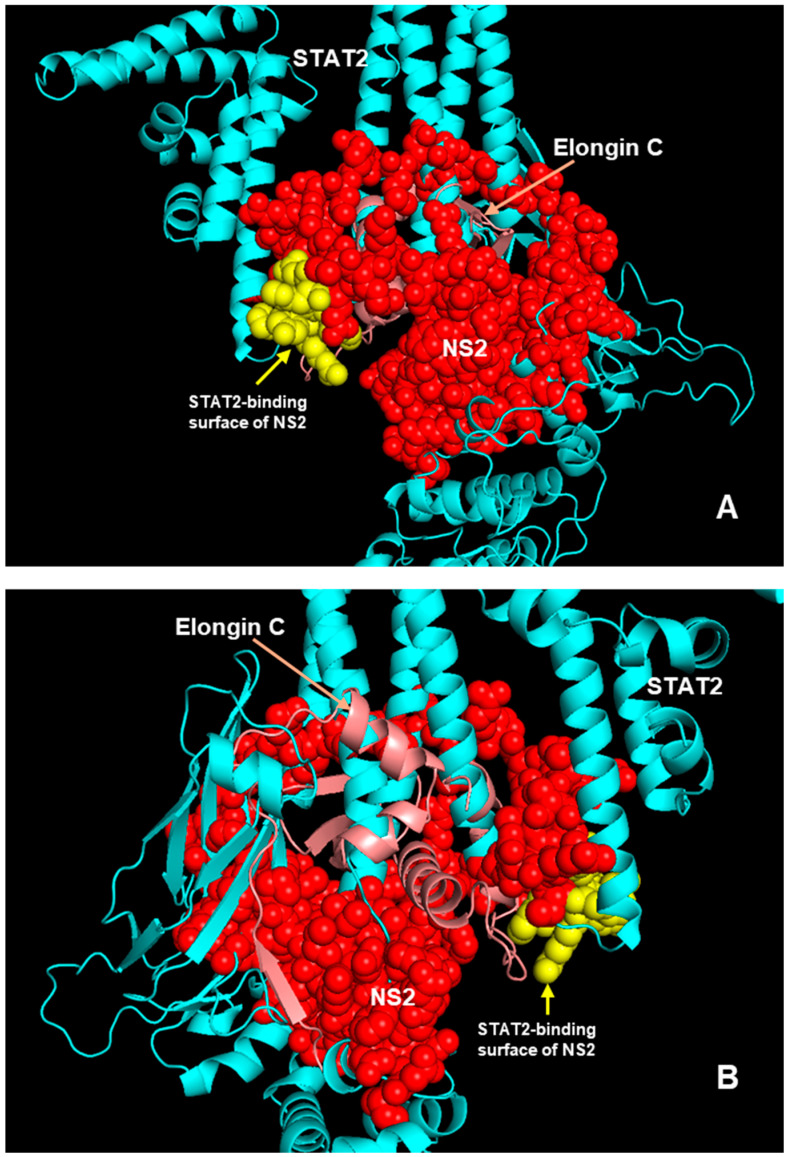
An enlarged view of the middle portion of the structures in Figure 9. (**A**,**B**) are rotated views of each other.

The central area of the model, where all the interactions are located (Figure 6), is also magnified for a better resolution (Figure 10).

Interestingly, the sequence of NS2 (yellow spheres in Figure 8 and Figure 9) that is in closest contact with STAT2, i.e., HRFIYLINH, overlaps with the BC-box sequence (Figure 5). A closer look at the front and back view of the complex, and the magnified view, revealed that other sequences of NS2 interact with several other areas of STAT2. A detailed list of all the interacting amino acids and the bonds and energies is outside the scope of this paper; however, the extensive interactions indicate that the BC-box of NS2 may have an important role in this complex. Elongin B is not included in this presentation since it was not interacting with either NS2 or STAT2, at least at this stage of the complex, which was also noted in the actual structure of the VHL complex (PDB 1LM8; Figure 4). Overall, the modeling results thus far agreed with known structures and interactions of the UPS components.

### 2.5. Mapping the Major Interacting Residues of RSV NS2

**Figure 11:**
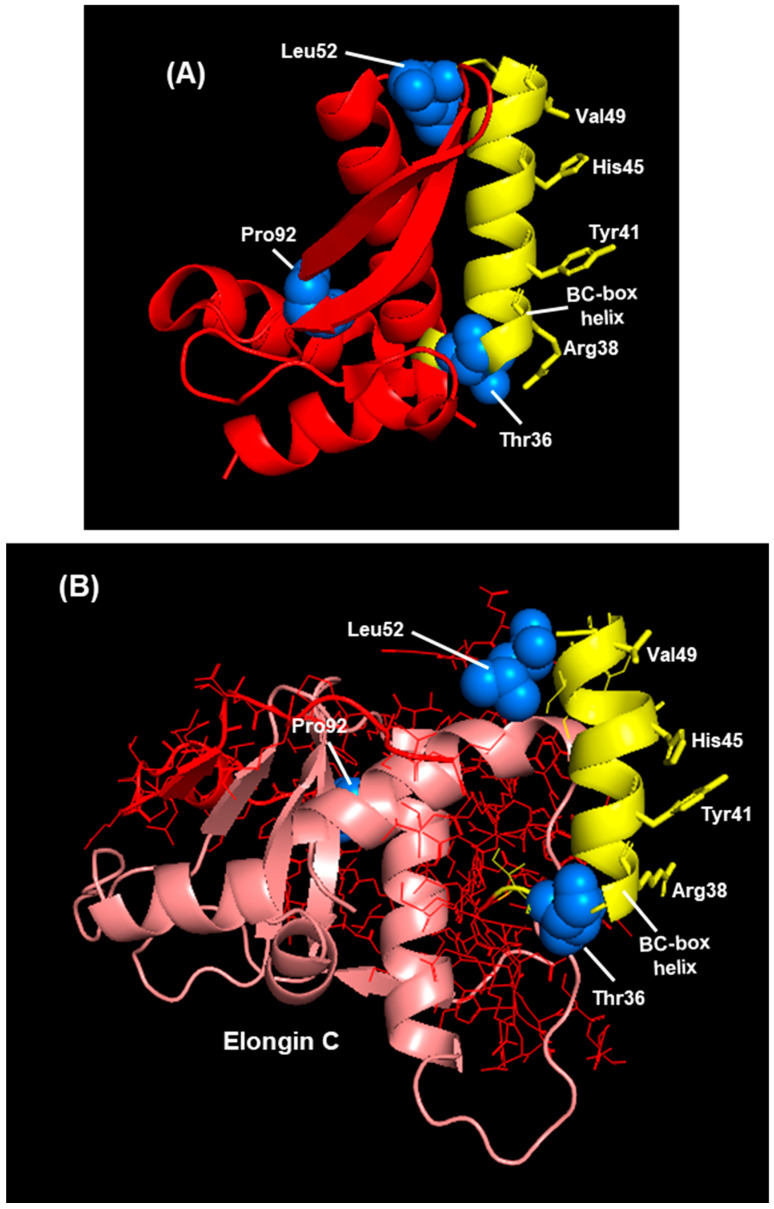
(**A**) RSV NS2 3D structure displayed in PyMol. Note that NS2 is the only protein in this panel and a few peripheral regions have been removed to focus attention to the relevant items that are labeled. While most of the NS2 structure is in red color, the BC-box is yellow. The three residues that are important for the ubiquitylating activity (T36, L52, P92) [16] are spheres of marine color. (**B**) A portion of the NS2-STAT2-Elongin C heterotrimer. This structure was presented in previous figures (Figure 9 and Figure 10) in a different orientation. Additionally, the STAT2 sequence has been completely removed here to offer an unobstructed view of the remaining structure. Lastly, the color codes used here are not meant to match those in Figure 8. In this panel, the NS2 color is the same as in panel (**A**), and Elongin C is in light pink. The structural representations are as follows: Nearly all of NS2 is in stick form, except the yellow and the blue areas, which are the same as in Panel (**A**). All of Elongin C is in cartoon form. In both panels, the side chains of the three resides (Arg, Tyr, His, Val), which are on the same face of the BC-box helix, are in stick form.

The prospect that the putative NS2 BC-box is involved in an interaction in the ligase complex encouraged us to investigate it further. In another development, Whelan et al. [16] reported a different set of NS2 amino acids that promotes ubiquitylation of host proteins. Using reverse genetic mutational analysis, the authors identified three residues in the amino-terminal half of NS2 that are essential for this activity: Thr36, Leu52, Pro92. When these residues were mutated, the ability of NS2 to degrade STAT2 was ablated [16], documenting the functional importance of this activity of NS2. Neither the structural aspects nor the mechanism of action of these three residues were explored further. Since the structure of NS2 is now determined and we are in search of a role of its BC-box-like motif, I located these residues in the 3D structures presented here to see if there is any structure–function correlation or interaction these two sets of residues in the 3D structure (Figure 11).

The readers may notice that the structure of NS2 in the two panels is slightly different in detail (e.g., slight distortion in the upper part of the yellow helix in panel B). This is because NS2 in panel A is an apo structure, not in complex with any other protein, whereas NS2 in panel B is in association with two other proteins (Elongin C, as shown, and STAT2). The interactions due to this association are expected to cause some structural distortions in all proteins to achieve the best fit for each. I now draw attention to several observations from the CSMs in this figure, not all of which may be discernible from this angle of view. First, the three ubiquitylation-promoting residues (blue spheres) of NS2 are spatially well-separated from one another. Second, nearly all of the BC-box has formed a single helix (yellow), which is mostly on the outer surface of the complex and is lined with three hydrophilic amino acid side chains (Arg, Tyr, His) that are relatively large and are not in contact with any structure. These amino acids are, therefore, solvent-exposed and available for interaction with a variety of molecules, while the interior face of the BC-box may make contact with Elongin C and/or STAT2. They may also bind portions of the large flexible loop of STAT2 (aa 701–783), whose flexibility may allow it to bend and reach the BC-box exterior. It is not known if any of the ubiquitylation-promoting amino acids are transiently ubiquitylated during transfer of the Ubq moiety to the substrates (e.g., STAT2). Nonetheless, Leu and Pro cannot link with Ubq because they lack the epsilon-amine group of a lysine (Lys) residue to which Ubq is typically attached. Thr can form an ester linkage with Ubq, but this is very rare [67]. I did not pursue the roles of these three residues any further.

The hydrophilic exterior face of the BC-box may also bind NS1 to form the heterodimer reported earlier [12]. Construction of a CSM for the NS1-NS2 heterodimer was not attempted, but this is a future goal. It is generally known that interdependency of the subunit interactions often stabilizes the whole complex [52]. It remains to be seen if attachment of the additional subunits, mentioned above, will make the CSM structurally more stable. It is also possible that the composition of the complex may differ between NS1 and NS2. In a previous experiment, in which broad deletions of recombinant STAT2 were tested for degradation by NS1 and NS2 in transfected cells, major differences were noted in the requirement of the various STAT2 domains by the two proteins [60].

**Figure 12:**
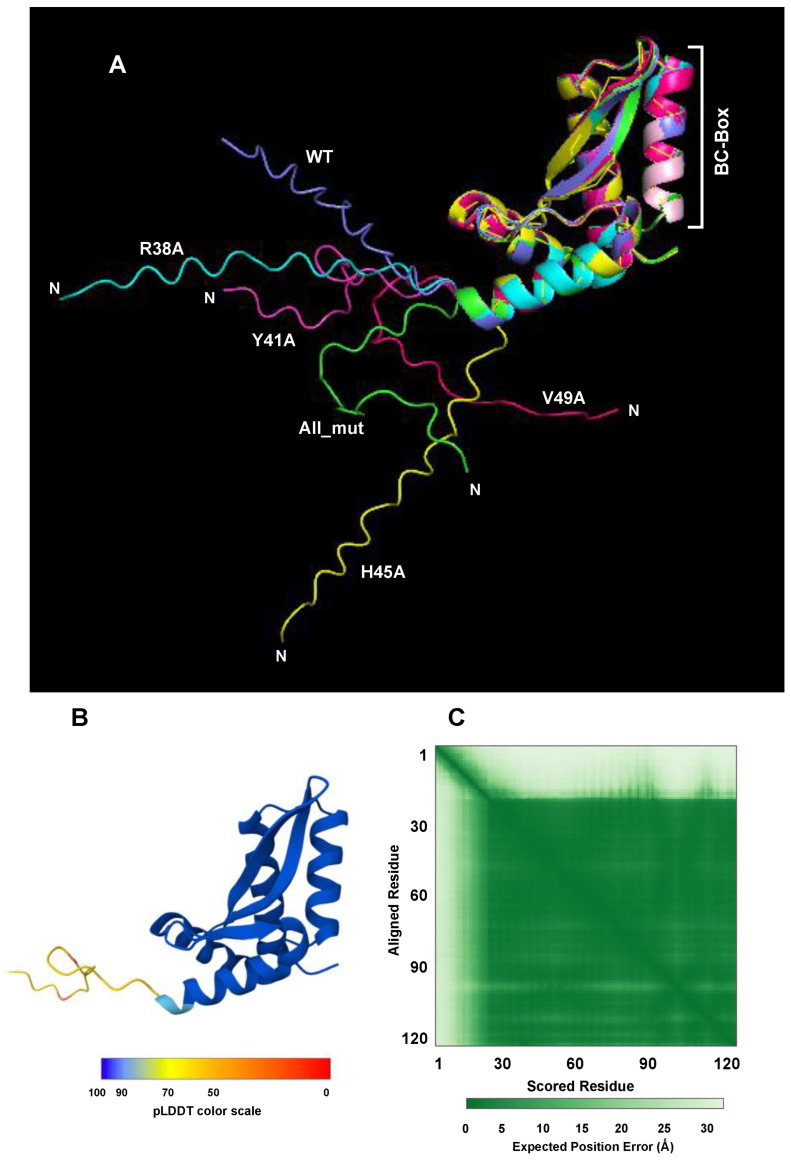
(**A**) Alignment of five BC-box mutant CSM with that of the wild type, displayed by PyMol. The CSM of the mutants are named by standard convention, such as Arg38 changed to Ala (R38A). WT is wildtype, and “All-mut” means all four amino acids were changed to Ala. Each structure is color-coded, as shown. Note that all six structures including the BC-box (labeled) are fully aligned, with the exception of the approximately ~22 amino acids at the very N-terminus. (**B**) The AF3 confidence level and (**C**) the alignment score plot of a representative structure of the BC-Box mutants as described in panel (**A**). The structure is colored by standard pLDDT color scale. Since all mutants had identical structures (panel (**A**)) and their confidence plots were also indistinguishable, only a random one (Y41A) is shown as representative of all.

To further ascertain the reliability of the NS2 structure in the previous Figures, I tested if the four amino acids, viz. Arg38, Tyr41, His45 and Val49, are truly on the exterior. I reasoned that if they are, their mutations may not affect the NS2 structure, but only abrogate external interactions with other proteins (which are not present in our structure). The four residues were changed individually and together in silico and their AF3-predicted structures were examined. Alignment of the structures indeed revealed that they were all identical and the same as the wild type (Figure 12).

What immediately stands out in all panels of Figure 12 is the nonalignment of the N-terminal segment of ~22 amino acids, the region that is also unstructured. This is because AF3 produces low-confidence predictions for unstructured flexible regions [68], and presents them in different orientations. In other words, AF3 essentially refuses to make a single fixed model for them.

The high AF3 confidence of the structure (pTM range 0.76–0.78 in the Panel B presentation) is in sharp contrast to the poor confidence (pTM ~0.64) in the previous predictions of NS2 that we grew accustomed to. The reason for this is rooted in the way AF3 is designed to explore the total structural landscape of a protein, which is presented in five models in its output package. Of the five, I routinely choose the one that receives the highest confidence of AF3, which AF3 designates ‘model 0’ and/or ‘best model’. The crystal structure, on the other hand, is biased towards one conformation that is the easiest to crystallize, which may not represent any of the AF3 models. As a result, AF3 often shows low confidence for the crystal structure, but high confidence in its own ‘best model’. All the previous NS2 structures were the crystal structure from 7LDK, which explains the low AF3 confidence. In the BC-box mutagenesis analysis, in contrast, we started with the full-length amino acid sequence and left it for AF3 to fold them, since the crystal structure is not important here, but rather we wanted to see if AF3 folds it differently, which may reveal something novel about the BC-box structure.

The apparent failure of all BC-box mutants (including the quadruple mutant ‘All_mut’) to alter the structure suggests the following scenarios: (a) These side chains are facing outward and, thus, do not interact with the force-field of the rest of the protein. (b) They could still be important for interaction with other proteins of the UPS complex that we do not know of. Only future studies can reveal what binds to this box. In either scenario, we can at least conclude that the helical structure is likely correct since it positions all four side chains on the exterior face.

## 3. Discussion

The structure predictions implicate roles of multiple amino acid residues of the large STAT2 protein in binding NS1 and NS2 (Figure 1 and Figure 2). The finding that they are far apart in the STAT2 structure (Figure 4) is in harmony with the dissimilarity between NS1 and NS2 in both sequence and structure. It also agrees with the functional analysis of recombinantly expressed chimera of human and mouse STAT2, which showed that a single domain of STAT2 is not sufficient for NS1/NS2-mediated degradation. However, degradation results using the interspecies chimera may have limited value since mouse and human STAT2 are significantly divergent not only in length but also in their modest sequence identity of ~70%. No systemic mutational analyses of human STAT2 were done to identify all the residues that are essential for IFN suppression or NS2 binding.

The consistently observed low confidence of AF3 in RSV NS1 and NS2 structures (e.g., pLDDT < 50) is most likely due to the following reasons that are built into the very algorithm of AlphaFold, which was already discussed in brief for Figure 12. The other reason may be that AF3 most heavily relies on multiple sequence alignment (MSA) of orthologous proteins with a good balance of similar and dissimilar residues [69]. AF3 did find NS sequences of many RSV isolates, but they were virtually identical, lacking the breadth of evolutionary information. Finally, so far, only one crystal structure is available each for NS1 and NS2, and, thus, AF3 cannot infer structural patterns because NS is poorly represented in the PDB. In general, if a protein lacks close homologs with known structures, its prediction confidence drops significantly. When RSV was discovered, it was found to be so unique that a new genus (*Pneumovirus*) was created for it. With the discovery of PVM, the *Pneumovirus* genus found its only other member; however, the PVM nonstructural proteins were very different from those of RSV [19,70], and thus could not be useful for the MSA requirement of AF3.

Due to the lack of appropriate resources, I have not been able to use molecular dynamics simulations to determine the real-time stability and structural variation in the complexes at an atomistic level [71]. However, the dissociation constants (Kd) predicted for the CSMs created here generally agreed with the known efficacy and comparative stability of two homooligomers. The real-life relevance of this comparison, if any, will require the quantification of the relative concentrations of NS2 and NS1 in the RSV-infected cell, which is not a trivial task and remains to be done. Regardless, the two NS proteins may compete for binding and degrading STAT2. This is an important consideration in actual RSV infection as it is also related to the half-life of the two NS proteins. It is known that when NS is limiting, STAT2 degradation cannot be detected [60,72]. The different efficiency of degradation of the other substrates could be due to the quantitative difference in the strength of interaction between amino acid side-chains, and/or the precision of structural fit. CMS constructions may provide an early direction to these alternatives.

Viral subversion of IFN response by the UPS-based mechanism is a remarkable example of host–pathogen interaction that has evolved to balance the interests of the pathogen and the host. While a role of NS proteins in the destruction of STAT2 is proven, an unsolved mystery is how the NS proteins of RSV destroy/inhibit a large repertoire of IFN pathway proteins besides STAT2. RSV NS2 degrades mostly STAT2, but RSV NS1 degrades several others, most efficiently RIG-I, TRAF3, IKKε, TRIF, and IRF7 [72]. Neither protein affects STAT1 levels. However, they were still specific in that most of the other IFN pathway proteins were unaffected. As we discussed elsewhere, the molecular mechanism of this “promiscuity versus specificity” remains unclear [60]. Docking structures, created with the different substrates and non-substrate controls, may provide insights into this intriguing question.

Based on the UPS complex models presented here, I would like to postulate a structural mechanism, founded on the “substrate promiscuity” of CRLs. The human genome encodes ~600 E3 ligases, in which the largest family is the Cullin-RING (CRL) E3 ligases that are assembled combinatorially from seven different Cullin scaffolds [60,73,74]. The E3 ligase, the final enzyme in the ubiquitylation cascade, can actually target multiple substrates. In analogy to enzymology, we can call this “substrate promiscuity”. A notable example is the F-box protein Cdc4 of yeast that regulates multiple processes by recruiting various cell cycle inhibitors and several other proteins for UPS-mediated degradation [74]. Likewise, the NS proteins may recruit different CRLs for different substrates. In corollary, the virally assembled UPS complexes, of which the NS proteins are the cardinal components, have the structural flexibility to accommodate the domains of several substrates. I presume that these interacting domains in the substrates have some degree of structural commonality; for example, human RIG-I, TRAF3, IKKε, TRIF, and IRF7 may have one or more motifs that are similar and bind to the RSV NS1-E3 ligase complex. By the same reasoning, RSV NS2 may recruit a different CRL subunit, assembling a ligase complex whose substrate-interacting groove fits STAT2 in preference to any other protein. The disordered regions of the proteins, which could not be modeled, may allow for plasticity and dynamism in the interactions [75].

**Figure 13:**
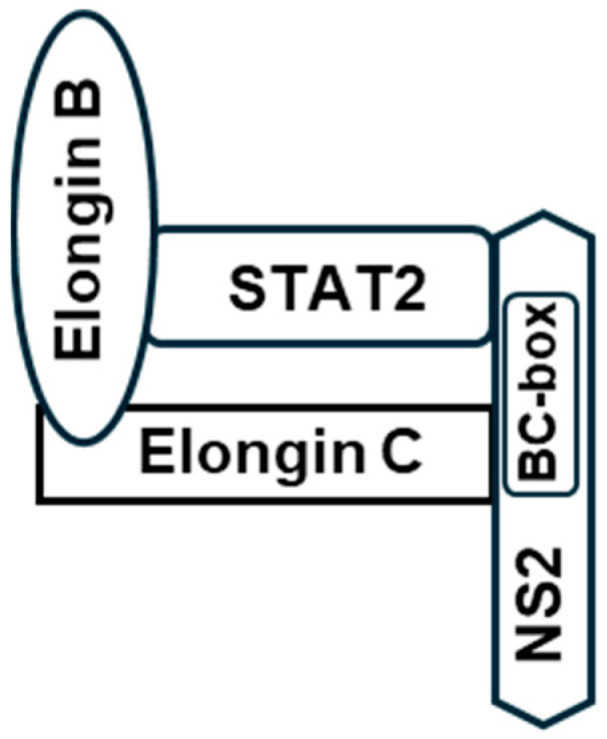
Summary model for the NS2 complex. This is a STAT-centric view of “our area of focus” shown in Figure 7, based on 3D structures presented in this paper, mainly in Figure 8 and Figure 9. It is also rooted in the VHL E3 ligase complex mentioned earlier. Here, the NS2 BC-box is shown to make contact with Elongin C and STAT2, while Elongin B binds to the N-terminal end of Elongin C [62]. Thus, the BC-box amino acids that are on the interior of the complex (a portion of the yellow helix in Figure 8) can be postulated to be in contact with Elongin C, whereas those on the outside of the complex (Arg, Tyr, His in Figure 8) are available to make stabilizing contacts with other proteins. Also, note that this is a highly simplified schematic, and in reality, essentially all subunits may interact with one another through multiple contact points of different strengths.

Most of these questions can be answered by existing structural and biochemical techniques. These may include structure-matching searches such as DALI [76], FoldSeek [77] and jFATCAT [78,79], and matching programs that are integrated with visualization tools, such as UCSF Chimera/ChimeraX [80] and PyMol [38], which was used in several studies here. In wet lab experiments, immunoprecipitation or affinity purification using tagged STAT proteins, followed by Mass Spec analysis, may reveal the identity of all the CRLs in the NS-transfected cell extract. Such studies with aramyxoviral IFN suppressor V proteins revealed the Double-stranded DNA-Binding Protein (DDB1) as a subunit of the viral STAT2-degradation complex [81,82,83,84]. It will be interesting to know whether Elongin A, Elongin B, DDB1 proteins or other novel host proteins are detected in cross-linked NS-tagged complexes. Previously, a comprehensive proteomic analysis revealed that NS1 co-precipitates with ~200 cellular proteins, which included many nuclear proteins and complexes, such as cyclin C, mediator complex, and ATR [77] that are involved in cell cycle regulation and DNA repair. In agreement with this, NS1 was shown to have a role in the G1-phase arrest of the cell cycle [85], and RSV-infected A549 cells are enriched in the G0/G1-phase [86]. Intriguingly, NS1 also co-precipitated with RNA pol II, and Elongins (known as SIII) were originally discovered as regulators of the elongation function of RNA pol II [87]. How these interactions may dovetail with NS1 IFN suppression function and whether these proteins compete for a common domain of NS1 are fascinating queries of the NS1 mechanism. Such proteomic studies are yet to be done with NS2; nonetheless, based on its helical BC-box structure (Figure 7) and the ability to degrade STAT2, a model is proposed for the NS2 complex (Figure 13).

### Limitations of This Study

The four major limitations of this study are as follows:

(1) Bioinformatic modeling: The research described here is based almost exclusively on in silico modeling and docking performed by various programs. Given this caveat, the programs are modern, updated and developed through state-of-the-art AI and machine learning procedures that have been validated with experimentally obtained structures, through repeated training and improvements. In essence, until an actual structure is available, the CSM will remain our best tool.

(2) Mutations of the BC-box residues: Our choice of Ala as the replacement for the BC-box amino acids is somewhat arbitrary. It is difficult to determine the best replacement amino acid for each since any change may cause unwanted effects, and regardless of the result, alternative explanations will be possible. For example, if the goal is to avoid interaction with another amino acid, than Gly could be a better choice because its “side chain” H is smaller than the -CH_3_ group of Ala. However, Gly will cause a kink in the alpha helix and throw other side chains off their contacts. A more detailed analysis of the NS2 and NS1 BC-Boxes is our future goal.

(3) Use of alternative modeling programs, other than AlphaFold: It is now well-known that the essence of AI, sponsored by Google DeepMind, was rapidly translated into the AlphaFold program, which has revolutionized protein structure prediction. In our studies, I have used AF multimer and PRODIGY for obtaining 3D structures well as thermodynamic binding affinity values (such as Kd/ΔG). Nonetheless, AlphaFold-Multimer can sometimes produce “forced” interactions in disordered regions. In such cases, other modeling and docking programs such as ClusPro (https://cluspro.org) [88], HADDOCK (https://wenmr.science.uu.nl/haddock2.4/) [89], Boltz-2 (https://app.tamarind.bio/boltz) [90] may be able to provide independent confirmation.

(4) Unhelpful RMSD: Although an overall low RMSD value is a good indicator of close alignment, it was not very useful for the NS complexes because of the low-confidence (pLDDT) scores of the NS2 part of the complex. Comparing two unreliable models would produce an arbitrary RMSD value that would not reflect the biological reality of the NS2 complex.

## 4. Materials and Methods

### 4.1. Materials

All studies were performed in silico by bioinformatic and mostly AI-based programs that are available in the public domain and are described in detail in the citations throughout this paper and in the corresponding references.

### 4.2. Methods

Most operations were conducted by AlphaFold version 3 (AlphaFold 3, often abbreviated as AF3 here). For generating complexes of two or more proteins (e.g., NS2-STAT2 or NS2-STAT2-EloC), AlphaFold3-Multimer was used at UCSF Chimera X (San Franciso, CA, USA) (https://www.cgl.ucsf.edu/chimerax/). AF3-Multimer provides metrics for evaluating the accuracy of its predictions: the predicted template modeling (pTM) score and the interface predicted template modeling (ipTM) score [65]. As mentioned before, the “best model” or “model_0”, which is ranked #1 among the five models in the output package, was used in all modeling studies because the ranking is based on structural confidence (pTM, ipTM). When using a RCSB-deposited PDB structure (such as NS1 or NS2) to complex with a AF3-folded structure, the ‘templates’ field in the AF3 input JSON was used to guide the folding process, if so needed. For independent confirmation, I sometimes used ClusPro (https://cluspro.org) [88] and Boltz-2 (https://app.tamarind.bio/boltz) [90], which are online resources.

The thermodynamic parameters, mainly Gibbs free energy change (ΔG) (related to dissociation constant, Kd), were derived by using PRODIGY (https://wenmr.science.uu.nl/prodigy/) [44]. All 3D structures were visualized and presented using PyMOL [38], and appropriate PyMol codes were used as needed.

## 5. Conclusions and Summary

The broad conclusion of this study is that properly selected and executed computations can lead to dependable 3D models that can provide independent corroboration of existing biochemical results as well as serve to triage future experimental plans. This has been possible largely because of improvements in structural modeling over the last decade, many incorporating artificial intelligence, machine learning, and neural networks, also known as ‘deep learning’, as well as, in a complementary approach, ab initio predictions and folding, exemplified by RoseTTAFold (https://neurosnap.ai/service/RoseTTAFold2) [33], which I did not use here. The reliability and usefulness of these bioinformatic approaches is underscored by the creation of a structural database within RCSB that houses over a million Computed Structure Models (CSMs) from sources such as the AlphaFold database (AlphafoldDB; https://alphafold.ebi.ac.uk/) (accessed on 1 December 2025) [31] and ModelArchive (https://modelarchive.org/) (accessed on 1 January 2026). The CSMs supplement the archive of experimental PDB entries, as I have shown in this study.

The specific results from our bioinformatic studies that pertain to the UPS complex assembled by RSV are as follows:

(a) Through methodical stepwise assembly, we observed the NS (nonstructural) proteins of RSV assembling the core UPS ligase complex that comprised the three key proteins: NS, STAT2, and Elongin C. These complexes corroborate the ability of the NS protein to destroy STAT2 by ubiquitylation and proteasomal digestion. They also revealed the interaction network of the amino acids.

(b) These results provide information about the NS-assembled core complexes that have proven too large and too difficult to crystallize.

(c) Although it was not possible to present the detailed image of each subunit embedded in the complex or the energy values of all side-chain interactions, these CSMs did provide usable information, which would be valuable when needed. To some extent, they also revealed the relative strength of interaction between any two proteins in the complexes.

(d) A promising new direction that emerged from these studies is the discovery that the NS2 BC-box may have a cardinal role in its UPS complex. Moreover, it is helical and its solvent-exposed outer face might interact with a protein that is yet to be discovered.

(e) Here, we have tested and optimized specific “docking” programs for the protein–protein complexes, which will be useful for future studies of trimeric complexes, and maybe even larger ones.

(f) Lastly, these AI models could serve as starting points for molecular dynamics simulations [69] that will capture the dynamic behavior of the NS2-STAT2 complex in full atomic detail and at fine temporal resolution.

## Data Availability

All data and related information are included in this article. The published PDB files can be and should be obtained from RCSB (rcsb.org) using the PDB numbers given. In addition, the structures in all figures have references for the programs used, which are also cited under Methods and in the References section. All protein sequences and thermodynamic values are presented in the Supplementary Materials.

## Supplementary Materials

The following supporting information can be downloaded at: https://www.mdpi.com/article/10.3390/ijms27052437/s1.

## Abbreviations

The following abbreviations are used in this manuscript:

AI	Artificial Intelligence
CSM	Computed Structure Model
UPS	Ubiquitin-mediated Proteasomal System
RSV	Respiratory Syncytial Virus
PVM	Pneumonia Virus of Mice
NS	Nonstructural
IFN	Interferon
STAT	Signal Transducer and Activator of Transcription
IRF	Interferon Regulatory Factor
ZIKV	Zika Virus
DENV	Dengue Virus
ISG	Interferon-Stimulated Gene
