# Supplementary material for: AI-Assisted Computed Structure Models for Pre-Ubiquitylation Complexes Assembled by Respiratory Syncytial Viral Suppressors of Cellular Interferon Response
Source: Int J Mol Sci. 2026 Mar 6;27(5):2437. doi: 10.3390/ijms27052437 (PMC12985925; doi:10.3390/ijms27052437)
Supplement: Supplementary file 1 [file ijms-27-02437-s001.zip › ijms-4134277-supplementary.pdf]

Supplementary material (Barik, IJMS)

**Part 1: Amino acid sequences and their sources**

>hSTAT2 (851aa); NP_005410.1; 6UX2 part underlined
MAQWEMLQNLDSPFQDQLHQLYSHSLLPVDIRQYLAVWIEDQNWQEAALGSDDSKATMLFFHFLDQLNYECGRCSQDPESLLLQHN
LRKFCRDIQPFSQDPTQLAEMIFNLLLEEKRILIQAQRAQLEQGEPVLETPVESQQHEIESRILDLRAMMEKLVKSISQLKDQQDV
FCFRYKIQAKGKTPSLDPHQTKEQKILQETLNELDKRRKEVLDASKALLGRLTTLIELLLPKLEEWKAQQQKACIRAPIDHGLEQL
ETWFTAGAKLLFHLRQLLKELKGLSCLVSYQDDPLTKGVDLRNAQVTELLQRLLHRAFVVETQPCMPQTPHRPLILKTGSKFTVRT
RLLVRLQEGNESLTVEVSIDRNPPQLQGFRKFNILTSNQKTLTPEKGQSQGLIWDFGYLTLVEQRSGGSGKGSNKGPLGVTEELHI
ISFTVKYTYQGLKQELKTDTLPVVIISNMNQLSIAWASVLWFNLLSPNLQNQQFFSNPPKAPWSLLGPALSWQFSSYVGRGLNSDQ
LSMLRNKLFGQNCRTEDPLLSWADFTKRESPPGKLPFWTWLDKILELVHDHLKDLWNDGRIMGFVSRSQERRLLKKTMSGTFLLRF
SESSEGGITCSWVEHQDDDKVLIYSVQPYTKEVLQSLPLTEIIRHYQLLTEENIPENPLRFLYPRIPRDEAFGCYYQEKVNLQERR
KYLKHRLIVVSNRQVDELQQPLELKPEPELESLELELGLVPEPELSLDLEPLLKAGLDLGPELESVLESTLEPVIEPTLCMVSQTV
PEPDQGPVSQPVPEPDLPCDLRHLNTEPMEIFRNCVKIEEIMPNGDPLLAGQNTVDEVYVSRPSHFYTDGPLMPSDF

>ZIKV NS5 (900aa); RdRP; YP_009227205
GTGNTGETLGEKWKNRLNALGKSEFQIYKKSGIQEVDRTLAKEGIKRGETDHHAVSRGSAKLRWFVERNLVTPEGKVVDLGCGRGG
WSYYCGGLKNVKEVKGLTKGGPGHEEPIPMSTYGWNLVRLQSGVDVFFTPPEKCDTLLCDIGESSPNPTVEAGRTLRVLNLVENWL
NNNTQFCIKVLNPYMPSVIEKMEALQRKYGGALVRNPLSRNSTHEMYWVSNASGNIVSSVNMISRMLINRFTMRHKKATYEPDVDL
GSGTRNIGIESETPNLDIIGKRIEKIKQEHETSWHYDQDHPYKTWAYHGSYETKQTGSASSMVNGVVRLLTKPWDIIPMVTQMAMT
DTTPFGQQRVFKEKVDTRTQEPKEGTKKLMKITAEWLWKELGKKKTPRMCTREEFTRKVRSNAALGAIFTDENKWKSAREAVEDSG
FWELVDKERNLHLEGKCETCVYNMMGKREKKLGEFGKAKGSRAIWYMWLGARFLEFEALGFLNEDHWFSRENSLSGVEGEGLHKLG
YILRDVSKKEGGAMYADDTAGWDTRITLEDLKNEEMVTNHMEGEHKKLAEAIFKLTYQNKVVRVQRPTPRGTVMDIISRRDQRGSG
QVVTYGLNTFTNMEAQLIRQMEGEGVFKSIQHLTVTEEIAVKNWLVRVGRERLSRMAISGDDCVVKPLDDRFASALTALNDMGKVR
KDIQQWEPSRGWNDWTQVPFCSHHFHELIMKDGRVLVVPCRNQDELIGRARISQGAGWSLRETACLGKSYAQMWSLMYFHRRDLRL
AANAICSAVPSHWVPTSRTTWSIHATHEWMTTEDMLTVWNRVWIQENPWMEDKTPVESWEEIPYLGKREDQWCGSLIGLTSRATWA
KNIQTAINQVRSLIGNEEYTDYMPSMKRFRREEEEAGVLW

>ZIKV NS5 (Chain B in 6UX2)
PNMKIIGRRIERIRNEHAETWFLDENHPYRTWAYHGSYEAPTQGSASSLVNGVVRLLSKPWDVVTGVTGIAMTDTTPYGQQRVFKE
KVDTRVPDPQEGTRQVMNIVSSWLWKELGKRKRPRVCTKEEFINKVRSNAALGAIFEEEKEWKTAVEAVNDPRFWALVDREREHHL
RGECHSCVYNMMGKREKKQGEFGKAKGSRAIWYMWLGARFLEFEALGFLNEDHWMGRENSGGGVEGLGLQRLGYILEEMNRAPGGK
MYADDTAGWDTRISKFDLENEALITNQMEEGHRTLALAVIKYTYQNKVVKVLRPAEGGKTVMDIISRQDQRGSGQVVTYALNTFTN
LVVQLIRNMEAEEVLEMQDLWLLRKPEKVTRWLQSNGWDRLKRMAVSGDDCVVKPIDDRFAHALRFLNDMGKVRKDTQEWKPSTGW
SNWEEVPFCSHHFNKLYLKDGRSIVVPCRHQDELIGRARVSPGAGWSIRETACLAKSYAQMWQLLYFHRRDLRLMANAICSAVPVD
WVPTGRTTWSIHGKGEWMTTEDMLMVWNRVWIEENDHMEDKTPVTKWTDIPYLGKREDLWCGSLIGHRPRTTWAENIKDTVNMVRR
IIGDEEKYMDYLSTQVRYLGEEGSTPGVL

RSV-NS1; AAA79091.1 (Putative BC-box is in RED)
MGSNSLSMIKVRLQNLFDNDEVALLKITCYTDKLIHLTNALAKAVIHTIKLNGIVFVHVITSSDICPNNNIVVKSNFTTMPVLQNG
GYIWEMMELTHCSQPNGLIDDNCEIKFSKKLSDSTMTNYMNQLSELLGFDLNP

RSV-NS2; AAA79090.1 (Putative BC-box is in RED; the Ubq-promoting sites are bold and underlined: T36, L52, P92; note that T36 and L52 are on the two ends of the BC-box.
MDTTHNDTTPQRLMITDMRPLSLETIITSLTRDII**T**HRFIYLINHECIVRK**L**DERQATFTFLVNYEMKLLHKVGSTKYKKYTEYNT
KYGTF**P**MPIFINHDGFLECIGIKPTKYTPIIYKYDLNP

>RSV-NS2 in 7LDK
MDTTHNDTTPQRLMITDMRPLSLETTITSLTRDIITHRFIYLINHECIVRKLDERQATFTFLVNYEMKLLHKVGSTKYKKYTEYNT
KYGTFPMPIFINHDGFLECIGIKPTKHTPIIYKYDLNP

>RSV-NS1 in 5VJ2
GHMGSNSLSMIKVRLQNLFDNDEVALLKITCYTDKLIHLTNALAKAVIHTIKLNGIVFVHVITSSDICPNNNIVVKSNFTTMPVLQ
NGGYIWEMMELTHCSQPNGLIDDNCEIKFSKKLSDSTMTNYMNQLSELLGFDLNP

>Human elongin-C; AAI00284.1

MDGEEKTYGGCEGPDAMYVKLISSDGHEFIVKREHALTSGTIKAMLSGPGQFAENETNEVNFREIPSHVLSKVCMYFTYKVRYTNS
STEIPEFPIAPEIALELLMAANFLDC

>PVM NS2 (156aa); AAW02833.1
MSTAMNKFTQTISKPATILNISDSEESGDEAGVGKVSRTTQSSERWLDLLIEKFQPSLQNITRYINWNFIRICNDRLKKEKMGYIE
AKQYVEDMAWMVIASEADSIEWKCIRRQEKVTGVKYPKFFFVQHKEDWIECTGCIPYPGHDLIYDEDDDD

>PVM NS1 (113 aa); AAW02832.1
MGCNVMMELDYGGRAAWLAFHITNFDRSDLETILRGARVCNTWQDQRLSVYLVGRDCNLLRPFVQAAKFIHNTRRGQTLTHWFTKN
IVFSSTGQETEPPIDPTCELLVELISG

<u>Sequences from crystal structure of the VHL E3 ligase complex (1LM8)</u>

>1LM8_4|Chain D|Hypoxia-inducible factor 1 alpha (**Hif-1α**)
DLDLEMLAPYIPMDDDFQLR
>1LM8_3|Chain C|**VHL** Von Hippel-Lindau disease tumor suppressor; the BC-Box motif is underlined
MEAGRPRPVLRSVNSREPSQVIFCNRSPRVVLPVWLNFDGEPQPYPTLPPGTGRRIHSYRGHLWLFRDAGTHDGLLVNQTELFVPS
LNVDGQPIFA<u>NITLPVYTLKERCLQVVR</u>SLVKPENYRRLDIVRSLYEDLEDHPNVQKDLERLTQERIAHQRMGD
>1LM8_2|Chain B|**ELONGIN C**|Homo sapiens; aa 17-50 needed to bind Elongin B:
MYVKLISSDGHEFIVKREHALTSGTIKAMLSGPGQFAENETNEVNFREIPSHVLSKVCMYFTYKVRYTNSSTEIPEFPIAPEIALE
LLMAANFLDC
>1LM8_1|Chain A|**ELONGIN B**|Homo sapiens
MDVFLMIRRHKTTIFTDAKESSTVFELKRIVEGILKRPPDEQRLYKDDQLLDDGKTLGECGFTSQTARPQAPATVGLAFRADDTFE
ALCIEPFSSPPELPDVMKPQDSGSSANEQAVQ

>RIG-I CTD (Chain A, Retinoic acid-inducible protein I, from *Anas platyrhynchos*, Mallard duck)
GAMGQKNLLCGKCKAYACSTDDIRIIKDSHHIVLGEAFKERYTTKPHKKPMQFDGFEKKSKMYCRNNNCQHDWGITVKYLTFDNLP
VIKIKSFVMESTATGTQMDFQKWKSINSSLKNFDVEEMSNLYPPF

>RIG-I CARD
EEYRLLLKRLQPEFKTRIIPTDIISDLSECLINQECEEILQICSTKGMMAGAEKLVECLLRSDKENWPKTLKLALEKERNKFSELW
IVE

**Part 2: Thermodynamic values of the 'complex' CSM (from AF3 and/ or PRODIGY)**

| CSM | Kd (M) | ΔG (kcal/ mol) |
|---|---|---|
| NS1-STAT2 | $1.2 \times 10^{-7}$ | -9.4 |
| NS2-STAT2 | $4.3 \times 10^{-11}$ | -14.1 |
| | | |
| NS2-RIG-I CTD | $3.1 \times 10^{-07}$ | |
| NS2-RIG-I CARD | $8.1 \times 10^{-10}$ | |
| | | |
| NS1-Elongin C | $9.7 \times 10^{-8}$ | |
| NS2-Elongin C | $9.3 \times 10^{-9}$ | |
| | | |
| Elongin C-NS2-STAT2 | $2.1 \times 10^{-10}$ | -13.2 |
| Elongin C-NS1-STAT2 | Not done | |
